# Non-Invasive Redox Biomarkers Detected in Organ Preservation Outflow Solution Enable Early Prediction of Human Liver Allograft Dysfunction

**DOI:** 10.3390/antiox14091104

**Published:** 2025-09-10

**Authors:** Daniel Vidal-Correoso, María José Caballero-Herrero, Ana M. Muñoz-Morales, Sandra V. Mateo, Marta Jover-Aguilar, Felipe Alconchel, Laura Martínez-Alarcón, Víctor López-López, Antonio Ríos-Zambudio, Pedro Cascales, José Antonio Pons, Pablo Ramírez, Kristine Stromsnes, Juan Gambini, Santiago Cuevas, Alberto Baroja-Mazo

**Affiliations:** 1 Molecular Inflammation Group, University Clinical Hospital Virgen de la Arrixaca, Biomedical Research Institute of Murcia (IMIB-Pascual Parrilla), 30120 Murcia, Spain; daniel.vidalc@um.es (D.V.-C.); mariajosefa.caballero@um.es (M.J.C.-H.); anamaria.munozm@um.es (A.M.M.-M.); sandra.valverde@imib.es (S.V.M.); marta.jover@imib.es (M.J.-A.); felipe.alchonchel@carm.es (F.A.); lma5@um.es (L.M.-A.); victor.lopez5@um.es (V.L.-L.); arzrios@um.es (A.R.-Z.); pedroantonio.cascales@um.es (P.C.); joseantonio.pons@um.es (J.A.P.); pablo.ramirez@carm.es (P.R.); 2 General Surgery and Abdominal Solid Organ Transplantation Unit, University Clinical Hospital Virgen de la Arrixaca, 30120 Murcia, Spain; 3 Hepatology and Liver Transplant Unit, University Clinical Hospital Virgen de la Arrixaca, 30120 Murcia, Spain; 4 Freshage Research Group, Department of Physiology, Faculty of Medicine, Institute of Health Research-INCLIVA, University of Valencia and CIBERFES, 46010 Valencia, Spain; kristine.stromsnes@uv.es (K.S.); juan.gambini@uv.es (J.G.)

**Keywords:** liver transplantation, donation after circulatory death, ischemia, oxidative stress, reactive oxygen species, biomarkers

## Abstract

Liver transplantation is commonly used for end-stage liver disease, but the demand for organs exceeds the supply, leading to the use of expanded criteria donors (ECDs). Organs from ECDs, especially from donors after circulatory death (DCD), encounter challenges like increased ischemia damage. Biomarkers, especially oxidative stress markers, may provide valuable insights for understanding and monitoring post-transplant events. Here, we highlight the unique value of organ preservation solution (OPS) as a non-invasive and early source of redox biomarkers, directly reflecting graft status during critical cold storage. This study investigated oxidative stress in 74 donated livers using OPS samples collected after cold storage, and also liver biopsies obtained before and after storage. We measured lipid peroxidation, protein carbonylation, DNA oxidation, and total antioxidant capacity from OPS, and performed gene expression analysis of liver biopsies. Oxidative stress markers differed based on donation type, with higher lipid peroxidation in DCD samples compared with donation after brain death (18.51 ± 2.77 vs. 11.03 ± 1.31 nmoles malondialdehyde (MDA)/mg protein; *p* = 0.049). Likewise, oxidative damage markers were associated with clinical outcomes: lipid peroxidation was increased in patients who developed biliary complications (21.86 ± 5.91 vs. 11.97 ± 1.12 nmol MDA/mg protein; *p* = 0.05), and protein carbonylation was elevated in those experiencing acute rejection (199.6 ± 22.02 vs. 141.6 ± 15.94 nmol carbonyl/mg protein; *p* = 0.005). Moreover, higher protein carbonylation levels showed a trend toward reduced survival (*p* = 0.091). Transcriptomic analysis revealed overexpression of genes associated with reactive oxygen species production in DCD livers. A predictive model for acute rejection integrating OPS biomarkers with clinical variables achieved 83% accuracy. Hence, this study underscores the importance of assessing oxidative stress status in preservation fluid as a biomarker for evaluating liver transplant outcomes and highlights the need for validation in larger, independent cohorts.

## 1. Introduction

Liver transplantation has become an essential treatment for patients suffering from terminal liver conditions, making it the second most common solid organ transplant procedure after kidney transplantation [1]. Despite advancements in surgical techniques and increased survival rates in recent years, the persistent high demand for liver allografts has exceeded their supply, necessitating the utilization of expanded criteria donors (ECDs) [2]. Liver allografts from ECDs are typically of lower quality, characterized by factors like advanced donor age, donors after circulatory death (DCD), or steatosis [3]. Such allografts are prone to acute ischemia–reperfusion injury (IRI), elevating the risk of early allograft dysfunction, primary non-function, thrombosis, ischemic-type biliary lesions, and other complications [2]. Despite these drawbacks, DCD has gained wider acceptance to meet the needs of medical centers [4]. Unlike donation after brain death (DBD), the procurement of DCD liver allografts takes place after circulatory arrest when resuscitation is no longer possible [4]. From that moment, warm ischemia begins and persists until the graft is flushed with preservation solution, leading to ATP depletion, oxygen deprivation, and generation of reactive oxygen species (ROS), particularly by hepatocytes [4,5]. Prolonged warm ischemia time (WIT) significantly impacts the quality of the liver allografts and surgical success [6]. The liver allograft is then stored at lower temperatures to reduce metabolic activity and preserve it until it reaches the hospital, a phase known as cold ischemia time (CIT), which is common in both DCD and DBD [7]. During CIT, hepatic sinusoidal endothelial cells are particularly affected, resulting in impaired microcirculation [8]. Finally, the liver allograft is implanted, restoring blood flow. The rapid increase in oxygen levels in ischemic tissue leads to ROS production, which, together with mitochondrial dysfunction and activation of innate immunity, constitutes the basis of ischemia–reperfusion injury [5,8]. IRI in the liver allograft may result in severe clinical consequences, including primary non-function, early allograft dysfunction, and biliary complications, all of which strongly affect graft and patient survival. Currently, specific parameters are employed to assess the condition of the liver allograft and determine its suitability for transplantation surgery [9]. Despite significant improvements in knowledge and technical aspects, there is a pressing need to gain a deeper understanding of what occurs during IRI and how it impacts the early post-transplant outcomes. Indeed, the first year after LT still carries a mortality rate of 10–15% [10] and graft loss rates ranging from 13 to 25% [11] highlighting the clinical impact of IRI. Acute rejection (AR), hepatic artery thrombosis, and biliary tract complications are among the most severe post-transplant adverse events, with incidence rates ranging from 2% to 35%, significantly contributing to both morbidity and mortality [11].

In this context, biomarkers offer an excellent opportunity to provide objective, measurable, and reproducible information on the state of the liver allograft [12]. However, most currently investigated biomarkers lack sufficient sensitivity or specificity in the early post-transplant period to guide clinical decision-making [13]. Given the continuous production of ROS by different cell lineages such as hepatocytes or Kupffer cells during IRI, oxidative stress markers represent promising candidates to closely reflect graft injury [14].

In this study, we assess oxidative stress markers encompassing lipids, proteins, and DNA in the organ preservation solution (OPS) of donated livers following static cold ischemic storage, aiming to ascertain their correlation with post-transplant events. Our findings reveal significant lipid peroxidation and protein carbonylation damage occurring during liver storage, potentially influencing transplantation outcomes as reflected in liver function parameters. Moreover, through logistic regression analysis, we identified recipient and donor age, donor body mass index (BMI), and markers of protein carbonylation, lipid peroxidation, and antioxidant capacity as the most predictive variables for the prediction of AR, achieving an accuracy of 83%.

## 2. Materials and Methods

### 2.1. Patients

All liver transplant recipients who received one of the studied donated livers participated in this study under written informed consent. The study received approval from the ethical committee of Hospital Clínico Universitario Virgen de la Arrixaca (2019-6-2-HCUVA) and adhered to the ethical principles outlined in the 1975 Declaration of Helsinki. A total of 74 adult patients were included for OPS collection between 1 July 2019 and 31 July 2022. Only patients whose samples could be processed and stored within the first 12 h by the IMIB-Biobank were included. Despite this restriction, our nonconsecutive sample cohort reasonably reflects the liver transplantation follow-up at our hospital [15]. Data were accessed for research purposes since 1 July 2019, and all demographic and clinical characteristics of donors and recipients were stored in an anonymized database in an electronic Case Report Form, and are summarized in Table 1. The study included 74 liver donors (62% donation-after-brain-death [DBD], 38% donation-after-circulatory-death [DCD], with DCD further stratified into super-rapid recovery [75%] and normothermic regional perfusion [25%]). Donors had a mean age of 60.5 ± 13.9 years (59.5% male), with comparable age (*p* = 0.952), sex distribution (*p* = 0.931), and cold ischemia time (328.5 ± 148.5 min; *p* = 0.287) across donation types. Functional warm ischemia time, defined as the interval between circulatory arrest and the initiation of cold flushing in the DCD protocol, did not differ between subgroups (*p* = 0.149). Recipients (mean age 57.4 ± 9.4 years; 64.9% male) predominantly had alcoholic cirrhosis (48.6%) or viral hepatitis (14.9%), while 13.5% required re-transplantation and 2.7% died intraoperatively. Donor causes of death included cerebrovascular accidents (67.4% DBD, 38.1% DCD-SRR) and anoxic encephalopathy (28.6% DCD-SRR); less frequent recipient etiologies comprised NASH and cryptogenic cirrhosis (4.1% each). Post-transplant immunosuppression was based mainly on tacrolimus, which was administered to more than 97% of patients.

### 2.2. Collection of Organ Preservation Solution

The OPS collection protocol can be found in a previous publication [16]. In brief, after static cold storage and before implantation, the infrahepatic inferior vena cava was ligated, and the liver grafts were flushed with 500 mL of 5% human albumin (Grifols, Barcelona, Spain) via the portal vein. Subsequently, the first 50 mL of intrahepatic end-ischemic OPS (eiOPS) was directly retrieved from the hepatic vein outflow into the suprahepatic inferior vena cava. The eiOPS was refrigerated, then centrifuged at 400× *g* for 10 min, and the supernatants were aliquoted and frozen at −80 °C until use.

### 2.3. Procurement of Liver Biopsies

In this study, an additional 36 donated livers were included for liver biopsy analysis (Table S1) [17]. Clinical and demographic characteristics were highly similar between cohorts, as detailed in Table S1. Liver tissue specimens were collected at two distinct phases of the transplantation process [17]. The T1 liver biopsies were obtained intraoperatively, directly in the organ procurement operating room at the time of organ retrieval, regardless of whether donation was via DBD or DCD. T2 biopsies were collected during bench surgery, immediately prior to implantation. All tissue samples were preserved in PAXgene Tissue Fixative containers (PreAnalytiX GmbH, Hombrechtikon, Switzerland) and subsequently processed for paraffin embedding.

### 2.4. Clinical Follow-Up of Transplant Patients

Our institutional protocol for post-transplant monitoring incorporates standardized surveillance methods, as referenced in prior publications [17,18]. Patients receive comprehensive biochemical monitoring, including daily liver function tests, during the initial postoperative week. Early graft function is assessed using the MEAF scoring system [9], incorporating peak ALT and INR values within the first 72 h along with day 3 bilirubin levels. Vascular evaluation includes weekly Doppler ultrasound assessments of hepatic vasculature (portal vein, hepatic artery, and hepatic veins) with flow velocity and resistive index measurements. For suspected hepatic artery thrombosis (prompted by abnormal liver tests, febrile episodes with bacteremia, or signs of cholangitis/sepsis), diagnostic confirmation involves repeat Doppler ultrasound followed by CT angiography evaluating the celiac axis and superior mesenteric artery. Biliary complications are investigated through hepatic ultrasound and MR cholangiopancreatography, with CT angiography additionally performed for non-anastomotic strictures. Acute rejection diagnosis requires ultrasound-guided liver biopsy interpreted according to Banff criteria [19]. One-year global post-transplant survival endpoints include either patient mortality or graft failure mandating retransplantation.

### 2.5. Total Antioxidant Capacity

The assay was performed using Total Antioxidant Capacity Assay Kit (Merck, Darmstadt, Germany, cat#MAK187). The experiment is based on the conversion of Cu^2+^ ions to Cu^+^ by the antioxidant molecules present in the samples. For each sample, a 5-fold dilution was carried out, and the assay was performed according to manufacturer’s instructions.

### 2.6. Lipid Peroxidation Analysis

Lipid peroxidation was determined as malondialdehyde (MDA) formation from lipoperoxides, which was detected using ultra-performance liquid chromatography (UPLC) as an MDA–thiobarbituric acid adduct following a previously described method [20]. This method is based on the hydrolysis of lipoperoxides in plasma and the subsequent formation of an adduct between thiobarbituric acid and MDA (thiobarbituric acid–MDA2). This adduct was detected using UPLC in reverse phase and quantified at 532 nm. The chromatographic technique was performed under isocratic conditions, with the mobile phase consisting of an aqueous mixture of 0.3% phosphoric acid and acetonitrile in an 80:20 ratio. The results were normalized by protein quantification.

### 2.7. Oxidative DNA Damage

The measurement was performed using OxiSelect^TM^ Oxidative DNA damage ELISA kit (Cell Biolabs Inc., San Diego, CA, USA, #cat STA-320), which is a competitive ELISA for the quantification of the oxidative DNA damage byproduct 8-hydroxydeoxyguanosine. Samples were diluted 10-fold, and the assay was performed according to manufacturer’s recommendations.

### 2.8. Protein Carbonyl Measurement

The quantification of carbonyl groups in proteins was carried out with Protein Carbonyl Content Assay Kit (Merck, cat#MAK094). A 10-fold dilution was performed for each sample.

### 2.9. Total Protein Quantification

The total protein concentration in each sample was measured using Pierce^TM^ BCA Protein Assay Kit (Thermo Fisher Scientific, Waltham, MA, USA, cat#23225).

### 2.10. Quantitative Reverse Transcriptase–Polymerase Chain Reaction (qRT-PCR)

RNA isolation from FFPE tissue was performed with the miRNeasy FFPE kit (Qiagen, Hilden, Germany, #cat 217504); the starting material was two 10 µm slides for each sample. qRT-PCR was performed using SYBR Premix ExTaq (Takara Bio Inc., Kusatsu, Japan, cat# RR420Q). Specific primers for *NRF2*, *HMOX1*, *NQO1*, *MFN1*, *BNIP3*, *FIS1*, *PINK1*, *NOX1*, and *NOX4* (Table S2) were purchased from Merck (KiCqStart^®^ SYBR^®^ Green Primers, #cat KSPQ12012, Merck). The samples were run in duplicate, and the relative gene expression levels were calculated using the 2^−ΔCt^ method, normalizing to 18S rRNA.

### 2.11. Statistical Analysis

Data were tested for normal distribution with the Shapiro–Wilk normality test. The homogeneity of data (homoscedasticity) was analyzed with the *F* test. A two-tailed unpaired *T* test for two-group comparison or ANOVA with the Bonferroni post-test for multiple group comparison was used wherever parametric testing applied (normal distribution and homoscedasticity), and the Mann–Whitney test or the Kruskal–Wallis test with the Dunn post-test was used when the dataset had to be analyzed nonparametrically. Correlation analyses were evaluated by using Spearman’s rank correlation. Survival analysis to assess the outcomes was performed using a survival curve, generated based on the Kaplan–Meier method, and the statistical significance of the differences between the survival curves was determined using the log-rank test. A logistic algorithm was applied for the generation of a predictive model with the language and environment for statistical computing R, version 4.4.2 (https://www.r-project.org/). Predictive variables were selected by the regularization and selection method LASSO (Least Absolute Shrinkage and Selection Operator) [21]. This process was complemented by a 5-fold cross-validation, which allowed the robustness and stability of the model to be assessed by varying the datasets over multiple iterations. The predictive model is then estimated training a Generalized Logistic Regression (GLM) model with these selected variables. This process involved splitting the dataset into 70% for training and 30% for testing. Subsequently, the confusion matrix is obtained to estimate the predictive ability metrics: sensitivity and specificity, balanced accuracy and Kappa index, as well as the area under de curve (AUC) and its 95% confidence interval. For the prediction model, missing values of predictor variables were imputed by the Classification and Regression Trees method [22], using the MICE library in R [23].

## 3. Results

### 3.1. Oxidative Stress Markers Exhibited Differences Based on the Type of Donation

**Figure 1:**
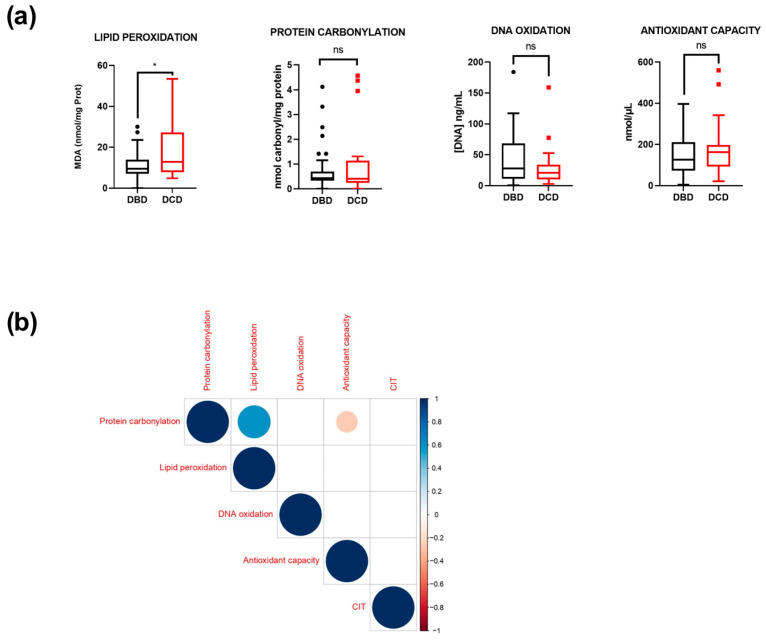
DCD livers exhibit increased oxidative damage: (**a**) Concentration of oxidative biomolecules in eiOPS from 74 explanted livers. Results are presented as medians, interquartile ranges, and minimum and maximum values. ns = not significant; * *p* ≤ 0.05. Data are presented as interquartile range and median. Outliers are shown as individual points. (**b**) Correlation matrix between cold ischemia time (CIT) and the concentration of oxidative biomolecules and total antioxidant capacity within eiOPS.

Throughout the liver transplantation process, organs are subjected to different forms of stress, impacting them through both direct and indirect pathways [24]. In order to elucidate the extent of oxidative damage in transplanted organs, we employed eiOPS as a non-invasive source and examined the oxidation of various biomolecules, including lipids, proteins and DNA (Table 2), reflecting an oxidative stress state. As discussed in the introduction, differences between DBDs and DCDs were evident at various stages of the transplantation process, such as the presence of warm ischemia in DCDs or uncontrolled death. A comparative analysis between DBDs and DCDs regarding each oxidized biomolecule and the total antioxidant capacity reflected increased lipid peroxidation in DCD samples (Figure 1a). Although other variables did not exhibit significant differences, a general trend to higher levels in DCDs was observed (Figure 1a). Additionally, the cause of death did not correlate with the oxidative stress detected in eiOPS.

Given the impact of redox imbalance on the presence of all biomolecules within cells, a correlation matrix was performed. As expected, two of the most prevalent biomolecules in cells, proteins and lipids, displayed a stronger positive correlation with oxidative form content (Figure 1b). Additionally, antioxidant capacity and protein carbonylation exhibited a negative correlation in this context (Figure 1b). Furthermore, considering that all organs are exposed to cold ischemia storage, a correlation test using CIT was deemed relevant. However, no significant correlation was identified (Figure 1b).

### 3.2. Transcriptomic Study Reveals the Influence of Donation Type on Genes Associated with ROS Production and Protection Against Oxidative Damage

**Figure 2:**
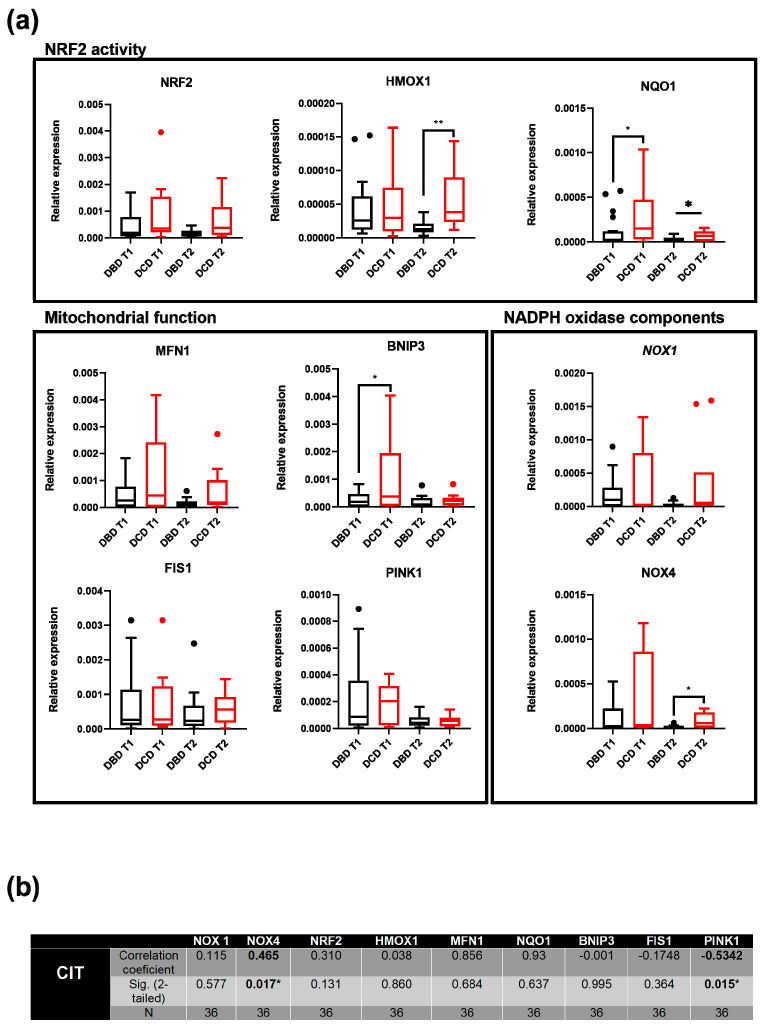
Transcriptomic analysis of oxidative stress-related genes: (**a**) Comparison of gene expression, as determined by qRT-PCR, between tissue biopsies obtained before liver procurement (T1) and after static cold ischemic storage (T2) in 36 donated livers, and categorized by donation type. * *p* ≤ 0.05; ** *p* ≤ 0.01. Data are presented as interquartile range and median. Outliers are shown as individual points. (**b**) Correlation matrix between CTI and the expression of different oxidative stress-related genes in T2 liver tissue samples.

It is worth noting that the impact of oxidative damage on an organ is not solely reflected in the oxidation of biomolecules; it also involves transcriptomic changes that unveil potential pathways related to redox restoration, ROS scavenging, or cell death in highly affected cells, among other processes [24]. Consequently, we selected several genes based on existing literature to compare their expression in biopsies obtained both before liver procurement (T1) and after static cold ischemic storage (T2). In this context, the comparison between DBD and DCD livers, both before and after cold ischemia, revealed that Heme oxygenase 1 (*HMOX1*), NADPH (nicotinamide adenine dinucleotide phosphate, reduced form) quinone dehydrogenase 1 (*NQO1*), BCL2 interacting protein 2 (*BNIP3*), and NADPH oxidase 4 (*NOX4*) were overexpressed in DCD samples (Figure 2a). While no differential expression was observed in the other examined genes (Figure 2a), it is noteworthy that all of them exhibited a tendency toward higher expression in DCD samples. Likewise, there was a significant decrease in gene expression in T2 samples when compared with T1 samples for almost all the genes analyzed (Figure S1).

Furthermore, a correlation analysis was conducted to assess the relationship between CIT and gene expression at T2 (Figure 2b). The results revealed a positive correlation between CIT and *NOX4* expression, while PTEN-induced kinase 1 (*PINK1*) expression exhibited a negative correlation. Once again, no correlation was identified between gene expression and the cause of death.

### 3.3. Oxidative Damage Impacts the Short-Term Outcome of Liver Transplant Patients

Oxidative stress is closely linked to ischemia–reperfusion injury during liver transplantation and various liver diseases resulting from impaired mitochondrial function, ultimately leading to cell death, inflammation, and fibrogenesis [24]. Therefore, monitoring redox alterations during the liver transplantation process could provide insights into potential pathologies that may develop after the procedure, allowing for the development of a biomarker signature for this purpose. We managed a one-year follow-up of 74 patients whose eiOPS was analyzed. The post-transplant course was marked by several complications, as outlined in Table 3. Among these, 23% of patients experienced AR, 8% faced hepatic arterial thrombosis, 16% suffered biliary complications, mainly strictures (8.1%), and 6 patients required re-transplantation due to graft loss. Unfortunately, 12 patients died within this timeframe.

**Figure 3:**
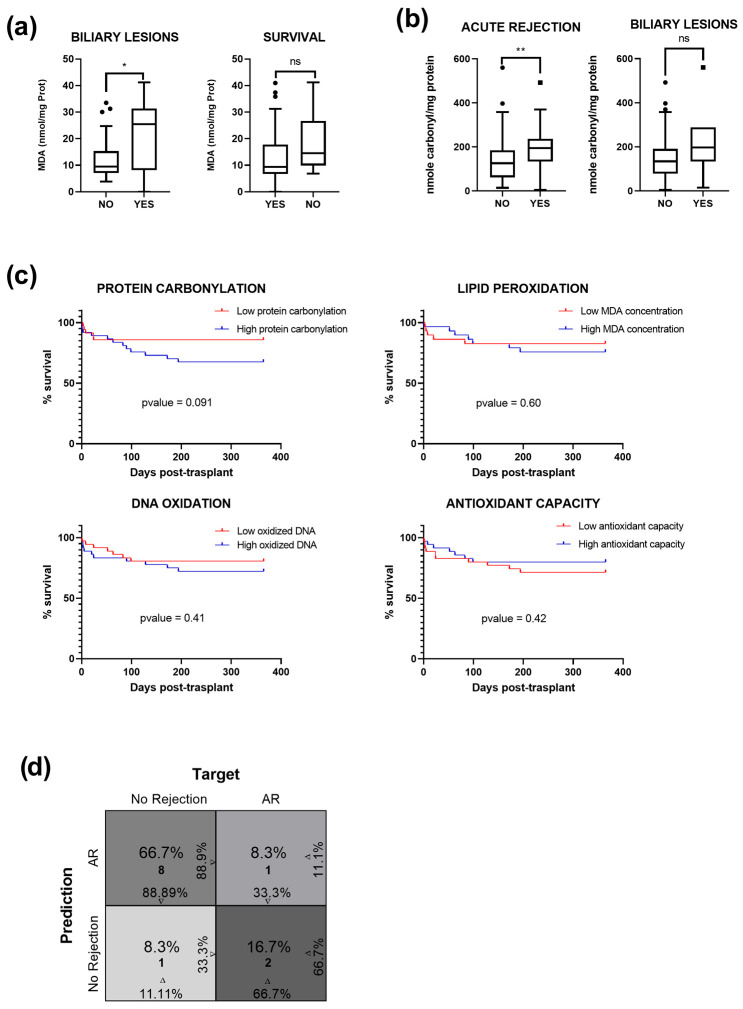
Patients developing pathology after transplantation experience larger oxidative damage: (**a**) Concentration of MDA quantified in eiOPS from 74 explanted livers, comparing individuals with and without biliary lesions, as well as patients who do or do not survive within the first year post-transplantation. Data are presented as interquartile range and median. Outliers are shown as individual points. (**b**) Concentration of protein carbonylation, comparing occurrence of acute rejection and biliary lesions in 74 patients. ns = not significant; * *p* ≤ 0.05; ** *p* ≤ 0.01. (**c**) Kaplan–Meier survival curves displaying the probability of survival for two different groups of patients sorted by the median concentration of each oxidized biomolecule and total antioxidant capacity. (**d**) Confusion matrix estimated from the predictive logistic model. The percentages on the horizontal axes of the first and fourth cells represent specificity and sensitivity, while the vertical axes show positive and negative predictive values, respectively. The relative percentages and counts for each cell are displayed at the center.

To establish an initial correlation, the concentration of each molecule was compared with the available clinical data. It was found that lipid peroxidation and protein carbonylation appeared to be strong indicators of the short-term progression of patients. Regarding MDA concentration, patients who experienced biliary lesions exhibited an increase in eiOPS (Figure 3a). Likewise, patients who did not survive displayed a tendency to have a higher concentration of lipid peroxidation in eiOPS (Figure 3a). On the other hand, an excess of carbonylated proteins may influence the development of AR (Figure 3b). Similarly, patients who experienced biliary injuries had a higher carbonyl content (Figure 3b). Having demonstrated a plausible association between oxidative damage and short-term outcomes, Kaplan–Meier curves were constructed (Figure 3c), dividing patients into two groups based on the median. The group with more pronounced signs of redox alterations exhibited a lower survival rate, although the difference did not reach statistical significance (Figure 3c). Conversely, antioxidant capacity showed the opposite trend (Figure 3c).

Moreover, we developed a predictive model for detecting the occurrence of AR. Utilizing a model composed of the studied biomarkers, along with several clinical and demographic variables (Table S3), the application of LASSO regression [21] identified seven key variables (Figure S2), including MEAF score [9], recipient and donor age, donor BMI, and the protein carbonylation, lipid peroxidation and antioxidant capacity, thus highlighting their relevance in the model. In the analysis of the GLM with variables selected by LASSO, we achieved an accuracy of 83% (95% CI: 0.5159–0.9791). Furthermore, the model exhibited a Kappa index of 0.5556. The sensitivity for positive cases (in this instance, ‘AR’) was 0.6667. In contrast, the specificity reached 0.8889, and the AUC demonstrated promising performance at 0.7777 (95% CI: 0.3288–0.9997) (Figure 3d and Table S4). Nevertheless, this model could not adapt to other post-transplant events due to their low incidence.

## 4. Discussion

In recent years, there has been growing interest in expanding the donor pool beyond the traditional DBD donations [25], leading to the reintroduction of DCD organ donation in many countries [4]. However, DCD donations typically entail longer WIT and higher complication rates compared to DBD grafts [26]. Efforts are underway to identify biomarkers to enhance understanding of donation mechanisms and improve organ quality. Prior studies conducted by our team have uncovered a heightened presence of various types of damage-associated molecular patterns [16,27], or extracellular vesicles [17] within eiOPS derived from DCD livers. The challenges of using systemic oxidative stress biomarkers in plasma or serum for clinical prediction are well-documented. Renal filtration and rapid redox turnover limit the accumulation of low-molecular-weight markers like MDA in circulation, particularly in patients with renal dysfunction or undergoing dialysis [28]. As a result, their clinical value as indicators of oxidative damage remains limited [29]. This contrasts sharply with our preservation fluid approach, where biomarkers reflect localized graft damage during ischemia, unaffected by systemic clearance mechanisms, providing a more direct and stable assessment of organ-specific oxidative stress. In our current investigation, we have similarly observed an increased concentration of oxidative stress markers in eiOPS derived from DCD livers compared to DBD livers. It is known that oxidative stress process is closely associated with IRI during liver transplantation, and it has been reported that MDA levels are elevated in cirrhotic and transplant patients and associated with hepatic damage [30,31]. MDA is a byproduct of lipid peroxidation, a process that occurs when free radicals interact with lipids in cell membranes [32,33]. Previous studies have indicated a transient increase in lipid peroxidation during liver transplantation, with oxidative stress markers gradually rising and peaking around one year post-transplantation. However, there was no clear association found between pre-transplantation urinary isoprostanes, formed from the free radical-catalyzed peroxidation of essential fatty acids, and clinical events such as AR [31]. We have determined several oxidative stress markers, including those related to protein, DNA and lipid oxidation, to evaluate the extent of oxidative stress in the transplanted organ. However, only MDA showed significant differences between groups, consistent with previous research [30,31]. As anticipated, lipid peroxidation levels were increased in livers from DCD donors compared with DBD. Extensive literature has documented oxidative stress and inflammatory damage in ischemia/reperfusion conditions [34,35], with MDA commonly employed as a marker to assess oxidative damage [14]. Our findings indicate an increase in *NOX4* expression among DCD patients, together with a positive correlation with CIT. This suggests that ischemia induced by cardiovascular death may trigger the upregulation of NADPH components, consequently leading to increased activity. NADPH oxidase activity increases ROS production and induces Nuclear factor erythroid 2-related factor (NRF2) activation, along with the expression of its target genes, such as *HMOX1* and *NQO1* [36]. On the other hand, oxidation products derived from certain endogenous lipids have been found to activate NLR family pyrin domain-containing 3 (NLRP3) inflammasome [37]. Hence, the accumulation of lipid peroxidation in the liver during ischemia–reperfusion process could contribute to the inflammatory response linked with AR [38]. Interestingly, biliary lesions were associated with the accumulation of liver MDA, suggesting that heightened oxidative stress could impair liver function and tissue integrity. However, despite this association, lipid peroxidation markers proved insufficient for predicting post-transplant events in our cohort of liver transplant patients.

Protein carbonylation, a form of protein oxidation catalyzed by excessive ROS production, leads to the formation of reactive ketones or aldehydes. Its occurrence has been linked to various conditions, including diabetic complications in type 2 diabetes, aging, and COPD, among others [39]. Our findings demonstrate an association between protein carbonylation and patient survival within our cohort. This observation suggests that protein carbonylation may serve as a useful biomarker for predicting graft loss and, consequently, the need for re-transplantation, as overall survival in our study was defined by both graft failure requiring re-transplantation and transplant-related mortality. Furthermore, this oxidative stress marker emerged as one of the variables with significant predictive value for detecting AR, alongside lipid peroxidation and antioxidant capacity, as well as demographic and clinical parameters such as the MEAF score. The MEAF score, which measures early allograft function ranging from 0 to 10 [9], has been independently validated [40] and predicts both recipient and graft survival. Furthermore, age and BMI are two factors strongly linked to oxidative stress and excessive ROS production [41], underscoring the importance of oxidative stress in predicting post-transplant events within our cohort of transplant patients.

Previous research has highlighted the potential of melatonin, a potent antioxidant, in mitigating steatohepatitis and enhancing outcomes following liver transplantation [42,43,44], emphasizing the significant role of ROS in these post-transplant complications and reinforcing the consistency of our findings. Furthermore, early investigations into antioxidant therapies in liver transplantation have suggested that interventions targeting mitochondria could effectively mitigate mitochondrial dysfunction and oxidative stress in hepatic pathogenesis, offering promise for improving patient outcomes [24]. Notably, our study revealed a negative correlation between CIT and *PINK1* expression. PINK1, a serine/threonine-protein kinase residing in mitochondria, is pivotal for maintaining mitochondrial quality control. It triggers mitophagy, a process entailing selective autophagy targeting mitochondria [45], and contributes to mitochondrial regeneration [46]. PINK1 deficiency has been associated with exacerbated liver injury [47], while its activation has shown protective effects against hepatic IRI [48], potentially mediated through NLRP3 inflammasome inhibition [49]. Moreover, we observed high expression of BNIP3, another protein involved in mitophagy [50], in DCD livers. However, certain genes associated with mitochondrial fusion and fission, such as mitofusin-1 (*MFN1*) and mitochondrial fission 1 protein (*FIS1*), did not exhibit differences.

Our findings of elevated oxidative stress markers in OPS align with established pathways of IRI, particularly NADPH oxidase activation and mitochondrial dysfunction [51]. NADPH serves as a crucial cofactor in multiple hepatic redox reactions. Among the enzymes utilizing NADPH, NADPH oxidase transfers electrons from NADPH to molecular oxygen, leading to the generation of ROS, primarily the superoxide anion. This process illustrates how ROS arise from the incomplete reduction of oxygen, producing partially reduced intermediates rather than their complete four-electron reduction to water [52]. Therefore, NADPH oxidase system, a key ROS generator in hypoxic conditions [53], correlates with our observed redox imbalance patterns, suggesting this pathway’s involvement in early graft damage even during cold storage. Clinically, this oxidative priming may predispose grafts to endothelial damage [54] and subsequent inflammation, as evidenced by our cohort’s increased incidence of early allograft dysfunction when redox thresholds were exceeded. This underscores the significance of assessing oxidative stress status as a biomarker for evaluating liver transplant outcomes [55].

While traditional IRI biomarkers focus on post-reperfusion tissue damage [56], our preservation fluid analysis identifies pre-transplant risk through non-invasive and cost-effective sampling, a critical advantage given the narrow therapeutic window for antioxidant interventions. This is supported by our prior work demonstrating that perfusate biomarkers (e.g., extracellular vesicles, damage-associated molecular patterns) predict early allograft dysfunction [16,17,27], reinforcing the clinical translatability of this strategy. Organ preservation techniques may contribute to oxidative stress, potentially impacting transplant outcomes [57]. Therefore, in addition to their role as biomarkers, boosting antioxidant defenses to maintain redox balance could prove pivotal in mitigating the damage produced by deceased donation and organ storage. Recent studies further support this paradigm, showing modified preservation solutions with antioxidants improve graft viability [58]. This strategy holds promise for enhancing the quality of marginal livers and improving liver graft outcomes. The synergistic approach of using postmortem normothermic regional perfusion (NRP), which involves cannulation of the femoral vasculature once death is declared in the hospital [59], to prevent warm ischemia, particularly in the case of DCD donors, alongside ex vivo normothermic machine perfusion (NMP) [60] to counteract static storage, presents an optimal scenario for the future of liver transplantation in the effective management of oxidative stress. NMP sustains organs at a physiological temperature, ensuring the delivery of oxygen and nutrients right up until the point of transplantation [61]. This technique also opens possibilities for administering therapeutic agents during the preservation phase of the liver, allowing for experimental drug testing in an ex vivo setting [62]. Additionally, NRP could be leveraged to administer treatments directly to donor organs, targeting oxidative stress at the source [63].

Nevertheless, the study does have limitations, as noted in previous publications [17,18]. Including NRP recipients introduces potential confounders, and while NRP has been proven to reduce mitochondrial injury [64] and lessen oxidative stress [65], our findings showed no discernible differences between NRP and super-rapid recovery techniques in DCD liver grafts. This may be attributed to the limited number of NRP donors in our sample. Likewise, and despite our efforts to increase the representativeness of biopsy analyses by including an additional 36 livers, we acknowledge that this could introduce potential confounding. Although Table S1 demonstrates there were no significant clinical or demographic differences between this and the primary cohort, except for the use of NRP, the inclusion of two partially overlapping patient sets remains a limitation of our study. Moreover, the unequal distribution of DBD and DCD donors, together with further subgroup stratification, adds heterogeneity that may reduce statistical power. As a result, some comparisons may be subject to type II error, and results should therefore be interpreted with caution. Additionally, the overall sample size was limited, and validation in new independent patient cohorts is critical to future integration of these findings into clinical practice.

## 5. Conclusions

This study highlights the potential of oxidative stress markers in the OPS as non-invasive indicators of liver graft viability and early post-transplant outcomes. Elevated levels of lipid peroxidation and protein carbonylation were associated with complications such as AR and reduced patient survival. These findings underscore the importance of redox balance during static cold storage and suggest that oxidative stress may contribute to post-transplant inflammation and injury. As a proof of concept, our work provides preliminary evidence that integrating these biomarkers with clinical parameters could support personalized risk stratification and guide optimized organ preservation strategies. However, our conclusions remain exploratory, and we explicitly acknowledge that further prospective validation in larger, multicenter cohorts is required before these biomarkers can be considered for routine clinical application. Such studies will also be essential to determine whether targeted antioxidant strategies may improve graft outcomes.

## Figures and Tables

**Table 1:** Demographic and clinical data of organ donors and recipients.

Variables	Donors (*n* = 74)			*p*	Recipients (*n* = 74)
	DBD (*n* = 46)	DCD (*n* = 28)			
		SRR (*n* = 21)	NRP (*n* = 7)		
**Age**	60.3 ± 13.6; 65 (22–87)	61.24 ± 12.6; 65 (30–77)	59.6 ± 21.6; 69 (26–82)	0.952 ^a^	57.4 ± 9.4; 58.5 (24–72)
**Sex**					
**Male**	27 (58.7)	13 (61.9)	4 (57.1)		48 (64.9)
**Female**	19 (41.3)	9 (38.1)	3 (42.9)	0.931 ^b^	26 (35.1)
**Body mass index**	25.5 ± 4.3; 25.1 (18.4–36.2)	25.5 ± 2.8; 25.4 (20.1–31.6)	28.0 ± 3.7; 28.6 (23.4–32.3)	0.269 ^a^	26.8 ± 4.2; 26.7 (18.7–36.8)
**CIT (min)**	312.7 ± 151.; 295 (90–960)	371.8 ± 137.3; 360 (180–630)	302.3 ± 153.4; 240 (150–540)	0.287 ^a^	
**Cause of death**				0.012 ^b^	
**CVA**	31 (67.4)	8 (38.1)	5 (71.4)		
**TBI**	10 (21.7)	4 (19)	1 (14.3)		
**Anoxic encephalopathy**	4 (8.7)	2 (9.5)	0 (0)		
**Cardiomyopathy**	0 (0)	6 (28.6)	0 (0)		
**Other**	1 (2.2)	1 (4.8)	1 (14.3)		
**Functional warm ischemia (min)**		17.6 ± 7.5; 17 (5–30)	13.1 ± 3.9; 15 (7–18)	0.149 ^c^	
**Diseases**					
**Alcoholic cirrhosis**					36 (48.6)
**Hepatitis C virus**					8 (10.8)
**Arterial thrombosis**					5 (6.8)
**Primary biliary cholangitis**					4 (5.4)
**MASH**					4 (5.4)
**Cryptogenetic Cirrhosis**					3 (4.1)
**Hepatitis B virus**					3 (4.1)
**Polycystic disease**					3 (4.1)
**Autoimmune hepatitis**					3 (4.1)
**Other**					5 (6.8)
**Re-transplant patients**					10 (13.5)
**Arterial thrombosis**					5 (50)
**Primary graft dysfunction**					1 (10)
**Cryptogenetic Cirrhosis**					1 (10)
**Ischemic cholangiopathy**					1 (10)
**Recurrent primary biliary cholangitis**					2 (20)
**Intraoperative death**					2 (2.7)
**Immunosuppressive treatment**					
**Tacrolimus**					72 (97.3)

Continuous variables are expressed as mean ± SD; median (range). Qualitative variables are expressed as frequency (%). CIT, cold ischemia time; CVA, acute cerebrovascular accident; TBI, traumatic brain injury; DBD, donation after brain death; DCD, donation after circulatory death; NRP, normothermic regional perfusion; SRR, super rapid recovery; MASH, metabolic dysfunction-associated steatohepatitis; LT, liver transplantation. ^a^ One-way ANOVA; ^b^ Fisher’s exact test; ^c^ *T*-test.

**Table 2:** Quantification of oxidative stress markers detected in eiOPS from 74 donated livers.

Oxidative Stress Marker	Concentration ^1^
Oxidized DNA (ng 8-OHdG/mL)	25.75 (0.58–183.4)
Lipid peroxidation (nmol MDA/mg prot)	8.81 (0–345.5)
Protein carbonylation (nmol carbonyl/mg prot)	0.44 (0–20)
Total antioxidant capacity (nmol/µL)	139.6 (0–560.4)

^1^ Variables are expressed as median (range). 8-OHdG, 8-hydroxydeoxyguanosine; MDA, malondialdehyde.

**Table 3:** One-year follow-up of the 74 liver transplant patients included in the eiOPS study.

Post-Transplant Event	Donation		*p*-Value
	DBD (46)	DCD (28)	
Acute rejection	10 (13.5)	7 (9.5)	0.632 ^a^
Hepatic arterial thrombosis	3 (4)	3 (4)	0.560 ^a^
Biliary complications			0.438 ^a^
Strictures	4 (5.4)	2 (2.7)	
Leaks	2 (2.7)	3 (4)	
Cholangitis	0 (0)	1 (1.4)	
Primary graft dysfunction	0 (0)	2 (2.7)	0.072 ^a^
Graft loss (re-transplantation)	2 (2.7)	4 (5.4)	0.145 ^a^
Deceased	7 (9.5)	5 (6.8)	0.829 ^a^
MEAF (Model for Early Allograft Function) score	2.95 (0.59–7.42)	4.14 (0.40–7.88)	0.041 ^b^

Continuous variables are expressed as median (range). Qualitative variables are expressed as frequency (%). ^a^ Chi-square test; ^b^ *T*-test.

## Data Availability

All datasets and protocols are available from the corresponding authors upon reasonable request to the corresponding author.

## Supplementary Materials

The following supporting information can be downloaded at https://www.mdpi.com/article/10.3390/antiox14091104/s1, Figure S1: Transcriptomic analysis of oxidative stress-related genes; Figure S2: Predictive variables for logistic model; Table S1: Demographic characteristics of organ donors included in the biopsy series; Table S2: Primer sequences used for qPCR; Table S3: Demographic and clinical variables included in the predictive model of acute rejection; Table S4: Performance Evaluation Metrics for Generalised Linear Regression Models (GLMs).

## Abbreviations

The following abbreviations are used in this manuscript:

AR	Acute rejection
AUC	Area under the curve
BMI	Body mass index
CIT	Cold ischemia time
CT	Computed tomography
DBD	Donor after brain death
DCD	Donor after circulatory death
ECD	Expanded criteria donors
eiOPS	End-ischemic organ preservation solution
GLM	Generalized logistic regression
IRI	Ischemia–reperfusion injury
LASSO	Least absolute shrinkage and selection operator
LT	Liver transplantation
MDA	Malondialdehyde
MEAF	Model for early allograft function
NLRP3	NLR family pyrin domain containing 3
NRP	Normothermic regional perfusion
OPS	Organ preservation solution
ROS	Reactive oxygen species
SRR	Super rapid recovery
WIT	Warm ischemia time
