# Supplementary material for: Non-Invasive Redox Biomarkers Detected in Organ Preservation Outflow Solution Enable Early Prediction of Human Liver Allograft Dysfunction
Source: Antioxidants (Basel). 2025 Sep 10;14(9):1104. doi: 10.3390/antiox14091104 (PMC12466377; doi:10.3390/antiox14091104)
Supplement: Supplementary file 1 [file antioxidants-14-01104-s001.zip › antioxidants-3807425-supplementary.pdf]

Oxidative stress state detected in organ preservation solution after cold ischemia as biomarker for liver transplantation

Daniel Vidal-Correoso, María José Caballero-Herrero, Ana M. Muñoz-Morales, Sandra V. Mateo, Marta Jover-Aguilar, Felipe Alconchel, Laura Martínez-Alarcón, Víctor López-López, Antonio Ríos-Zambudio, Pedro Cascales, José Antonio Pons, Pablo Ramírez, Kristine Stromsnes, Juan Gambini, Santiago Cuevas and Alberto Baroja-Mazo

Table S1.- Demographic characteristics of organ donors included in the biopsy series

**Variables**	**Donors (n = 36)**	***P*†**
Age	55.95 ± 14.82; 58 (16-77)	0.119 [a]
Sex		
Male	22 (61.1)	0.431[b]
Female	14 (38.9)	
Body mass index	26.32 ± 4.41; 25.71 (18.41-37.04)	0.463[a]
CIT (min)	269.50 ± 143.14; 240 (30-560)	0.055 [a]
Donation		
DBD	23 (63.9)	0.849[b]
DCD	13 (36.1)	
SRR	3 (23.1)	0.004[b]
NRP	10 (76.9)	
Cause of death		
CVA	17 (75)	0.188[b]
TBI	8 (22.2)	
Anoxic encephalopathy	4 (11.1)	
Cardiomyopathy	2 (5.6)	
Other	5 (13.9)	
Functional warm ischemia (min)	13.50 ± 6.49; 14 (2-25)	0.222[a]

Continuous variables are expressed as mean ± SD; median (range). Qualitative variables are expressed as frequency (%). CIT, cold ischemia time; DBD, donation after brain death; DCD, donation after circulatory death; NRP, normothermic regional perfusion; SRR, super rapid recovery; CVA, acute cerebrovascular accident; TBI, traumatic brain injury. †Comparison with the first 74 donations. [a]T-test. [b]Chi-square test.

Table S2.- Primer sequences used for qPCR

Gene	NCBI Gene ID	5´- Forward sequence	3´- Reverse sequence
NRF2	4780	GTCACTTGTTCCTGATATTCC	TCAGGAATGGATAATAGCTCC
HMOX1	3162	CAACAAAGTGCAAGATTCTG	TGCATTCACATGGCATAAAG
NQO1	1728	AGTATCCACAATAGCTGACG	TTTGTGGGTCTGTAGAAATG
MFN1	55669	ATCTTTGAGGAGTGTATCTCG	GTAGCTAGTATCTGTTTAGCTC
BNIP3	664	CAGTCTGAGGAAGATGATATTG	GTGTTTAAAGAGGAACTCCTTG
FIS1	51024	GGGATTACGTCTTCTACCTG	GAGTCCATCTTTCTTCATGG
PINK1	65018	CGTTATGAAGAACTATCCCTG	CAAGGATGTTGTCGGATTTC
NOX1	27035	CCGGTCATTCTTTATATCTGTC	CACCTTGGTAATCACAACC
NOX4	50507	AATTTAGATACCCACCCTCC	TCTGTGGAAAATTAGCTTGG

Table S3.- Demographic and clinical variables included in the predictive model of acute rejection

Demographic/clinical variable	Value
Donation	DBD/DCD
MEAF score	0 to 10
CIT	Minutes
Donor and Receptor age	Years
Donor and Receptor BMI	Ratio number
Donor and Receptor Sex	Male/Female

*DBD, donation after brain death; DCD, donation after cardiac death; MEAF, Model of early allograft failure; CIT, cold ischemia time; BMI, body mass index.

Table S4.- Performance Evaluation Metrics for Generalised Linear Regression Models (GLM)

RATING MODEL PERFORMANCE EVALUATION METRICS	VALUE
Accuracy	0.83
Kappa index	0.5556
Sensitivity	0.6667
Specificity	0.8889
AUC	0.7777

The table provides a comprehensive overview of the performance evaluation metrics applied to the Generalised Linear Regression (GLM) model in the context of our predictive study. These metrics, including Accuracy, Kappa, Sensitivity, Specificity and AUC (Area Under the ROC Curve), provide a comprehensive assessment of the model's ability to make predictions, tailored to the specific classification task. Each value in the table represents the specific performance of the GLM model on each metric, allowing for a detailed comparison and a holistic understanding of its effectiveness in the predictive task addressed in this study.

Supplementary Figure 1

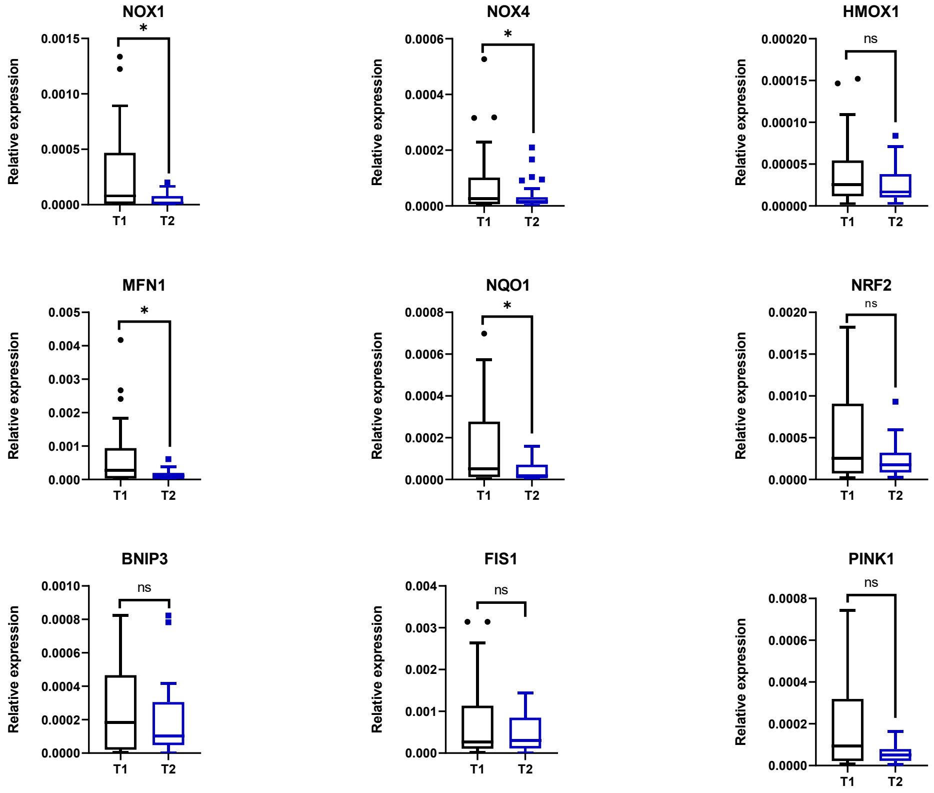

Figure S1.- Transcriptomic analysis of oxidative stress-related genes. Comparison of gene expression, as determined by qRT-PCR, between tissue biopsies obtained before liver procurement (T1) and after static cold ischemic storage (T2) in 36 donated livers.

Supplementary Figure 2

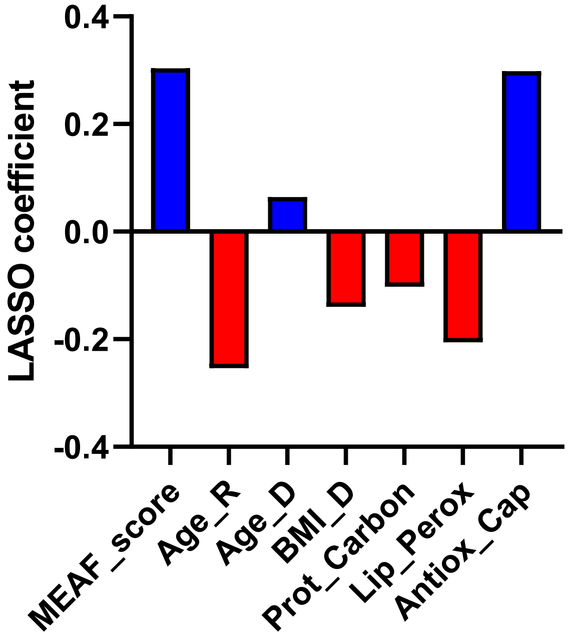

Figure S2.- Predictive variables for logistic model. Bar plot illustrating the coefficients estimated by the LASSO method (Y axis) for each candidate predictor (X axis).
